# Neuropeptide delivery to the brain: a von Willebrand factor signal peptide to direct neuropeptide secretion

**DOI:** 10.1186/1471-2202-11-94

**Published:** 2010-08-11

**Authors:** Marijke WA de Backer, Maike AD Brans, Mieneke CM Luijendijk, Keith M Garner, Dianne MA van den Heuvel, R Jeroen Pasterkamp, Roger AH Adan

**Affiliations:** 1 Rudolf Magnus Institute of Neuroscience, Department of Neuroscience and Pharmacology, Utrecht University Medical Centre Utrecht, Utrecht, the Netherlands

## Abstract

**Background:**

Multiple neuropeptides, sometimes with opposing functions, can be produced from one precursor gene. To study the roles of the different neuropeptides encoded by one large precursor we developed a method to overexpress minigenes and establish local secretion.

**Results:**

We fused the signal peptide from the Von Willebrand Factor (VWF) to a furin site followed by a processed form of the Agouti related protein (AgRP), AgRP_83-132 _or α-melanocyte stimulating hormone. *In vitro*, these minigenes were secreted and biologically active. Additionally, the proteins of the minigenes were not transported into projections of primary neurons, thereby ensuring local release. *In vivo *administration of VWF-AgRP_83-132 _, using an adeno-associated viral vector as a delivery vehicle, into the paraventricular hypothalamus increased body weight and food intake of these rats compared to rats which received a control vector.

**Conclusions:**

This study demonstrated that removal of the N-terminal part of full length AgRP and addition of a VWF signal peptide is a successful strategy to deliver neuropeptide minigenes to the brain and establish local neuropeptide secretion.

## Background

Numerous neuropeptide precusors have been implicated in the regulation of energy balance, such as pro-opiomelanocortin (POMC), melanin concentrating hormone (MCH) and ghrelin. Neuropeptide precursors have a propeptide which translocates the neuropeptide precursors from the rough-endoplasmatic reticulum to the trans-golgi network (TGN). From the TGN the peptide precursors traffic into immature secretory vesicles where they are cleaved once or multiple times by prohormone convertases (PC) 1/3 and/or 2 to generate functional neuropeptides [1-3]. Through budding and fusion the immature vesicles become mature, dense, vesicles which release their content upon a specific signal [4].

The processing of neuropeptide precursors results in multiple biologically active peptides, sometimes with opposing effects; an example is the POMC gene. Pre-pro POMC is processed into several neuropeptides with anorexigenic functions, e.g. α-melanocyte stimulating hormone (MSH) and β-MSH, but also into an opioid with "orexigenic" function, namely β-endorphin [5-9]. Loss of the whole POMC gene results in hyperphagia and obesity [10]. Although there is strong evidence that the lack of melanocortin signaling in POMC knockout mice explains the phenotype, mice lacking only the β-endorphin part of the POMC gene are also hyperphagic and obese [11]. Another example of a precursor gene that encodes for peptides with diverse effects is pre-pro-ghrelin which encodes for ghrelin, an appetite stimulating hormone, and obestatin, an appetite suppressing hormone [12]. Therefore, it is important to determine the role of individual neuropeptides encoded by a precursor gene.

To investigate the role of specific neuropeptides, synthesized neuropeptides can be injected through intracerebroventricular (ICV) or local cannulae in the brain. However, there are drawbacks to these techniques. ICV administration of neuropeptides can be done long term, but administration is not local. In contrast, neuropeptides remain local with local cannulae, but these experiments are usually short term, because the cannulae can not be maintained over weeks. To overexpress neuropeptides locally and for long periods of time one can use viral vectors, such as adeno-associated viral (AAV) vectors [13-15]. There are two problems with the long term overexpression of neuropeptide genes. Firstly, the precursor gene encodes for multiple peptides, which may serve different functions (see above). Therefore, overexpression of the precursor gene may show the effects multiple peptides. The second problem, with overexpression of neuropeptides via viral vectors, is that the neuropeptide is potentially released in an area to which the transduced neuron projects rather than secretion locally at the site of transduction; with AAV mediated overexpression of neuropeptide Y (NPY) in the paraventricular nucleus (PVN) [13,16] the release of NPY may thus not be limited to the PVN. In the hypothalamus there are many interneurons and local connections, therefore NPY will probably be secreted locally. However, even in the absence of staining in the projection areas one can not exclude that a projection neuron is releasing the neuropeptide elsewhere.

In order to circumvent that overexpression of a neuropeptide gene generates multiple messages and that the peptide may be released at distant sites, we developed an AAV vector based method to overcome these problems. In short, we fused a signal peptide with HA tag and furin site to the cDNA of a specific minigene, namely AgRP_83-132 _or α-MSH. To prevent trafficking of the minigene into the regulated secretion pathway we removed the N-terminal part of AgRP or only used the MSH sequence and made a construct in which a signal peptide from the Von Willebrand Factor (VWF) was used to enter the endoplasmic reticulum [17,18]. A furin cleavage site was inserted between the signal peptide-HA tag and the minigene to release the minigene from the signal peptide. Furin is a ubiquitously expressed PC which is located in the TGN, in stead of in secretory vesicles, and cycles back and forth to the plasma membrane [19-21]. We used the cDNA of AgRP_83-132 _as a minigene, because this is the main secreted form of AgRP after processing by PC [22]. AgRP is an inverse agonist for the MC3R and MC4R and stimulates food intake and reduces energy expenditure, while α-MSH is a peptide derived from the POMC gene and is able to activate the MC3R and MC4R to inhibit food intake and increase energy expenditure [5,23-26]. AgRP and α-MSH compete to bind to the MC4R and the balance between these peptides determines the activity of the MC4R.

## Results

### VWF-AgRP_83-132 _and VWF-αMSH are secreted and biologically active

To investigate if peptides produced from constructs with a VWF signal peptide followed by a cleavage site and a neuropeptide are secreted and are biologically active, we tested whether the medium of cells transfected with the VWF-AgRP_83-132 _constructs was able to antagonize the MC4R. The ability of the VWF-AgRP_83-132 _to shift the NDP-MSH dose reponse curve to the right was compared to a construct encoding for full length AgRP, containing the normal AgRP signal peptide. Activation of MC4R *in vitro *results in an increase in cyclic adenosine monophosphate (cAMP)[27]. In our assay upregulation of cAMP increased LacZ expression through a cAMP responsive element.

**Figure 1:**
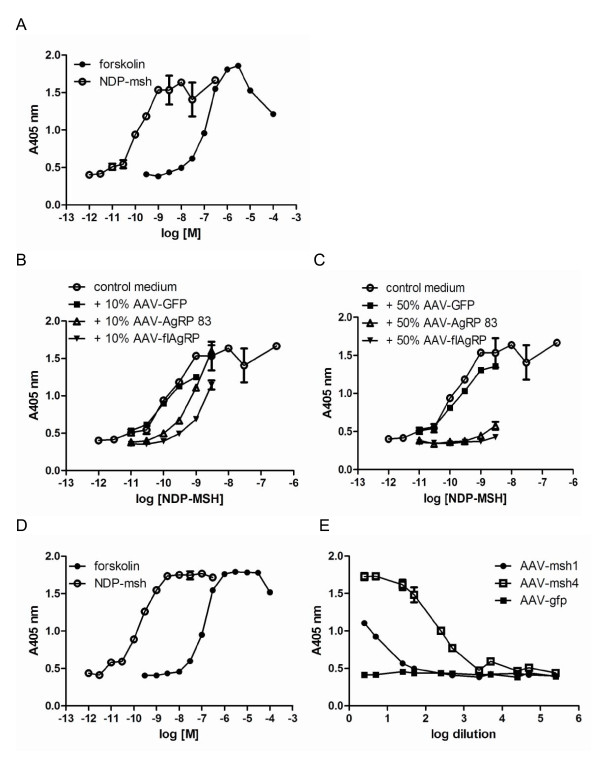
**VWF-proteins are secreted and biologically active**. A: Dose-response curve of NDP-MSH and forskolin showing their ability to stimulate 293T cells transfected with MC4R and cAMP sensitive reporter gene. B: similar as 2A, but in the presence of 10% of conditioned medium from 293T cells transfected with either AAV-GFP, AAV-flAgRP or AAV-VWF-AgRP_83-132 _. C: similar as 2B, but with 50% of conditioned medium. D: dose-response curve of NDP-MSH and forskolin as positive control for MC4R and CRE-LacZ transfection. E: response curve of cells transfected with MC4R and CRE-LacZ incubated with different dilutions of conditioned medium. Medium was taken from cells transfected with AAV-GFP, AAV-MSH1 or AAV-MSH4. Graphs represent an average of 4 trials.

Addition of NDP-MSH to MC4R and CRE-LacZ transfected cells resulted in a dose response curve with an EC_50 _value of 0.1 nM (1 × 10^-10^). Another control was the addition of forskolin to MC4R_Cre-LacZ transfected cells (Figure 1A). Forskolin directly activates adenylyl cyclase, thereby increasing cAMP (control for transfection of MC4R and CRE-LacZ). When supernatants of GFP transfected cells were added together with different concentrations of NPD-MSH to MC4R_CRE-LacZ transfected cells, the dose response curve did not alter. However, addition of supernatants of flAgRP or VWF-AgRP_83-132 _transfected cells shifted the NDP-MSH dose response curve to the right. The EC_50 _decreased to 1.4 and 0.5 nM, respectively when 10% of the volume added was supernatant (Figure 1B). When 50% of the volume added to the MC4R_CRE-LacZ cells was supernatant from flAgRP or VWF-AgRP_83-132 _the NDP-MSH dose response curve shifted even further to the right (Figure 1C). In order to determine the amount of secreted AgRP protein, supernatants were analyzed on a spot blot (data not shown). This blot showed that 18.2-fold less AgRP was secreted from the VWF-AgRP_83-132 _transfected cells compared to flAgRP transfected cells, even though the transfection efficiencies were similar.

To check if monomeric VWF-αMSH1 and polymeric VWF-αMSH4 were able to activate the MC4R, different dilutions of supernatants from cells transfected with these constructs were added to MC4R_CRE-LacZ transfected cells. Supernatants of both constructs activated the MC4R, with the polymeric VWF-αMSH4 being more potent. The polymeric VWF-αMSH4 reached 50% of MC4R activation at a dilution of 216 times, while VWF-αMSH1 already reached 50% activation of MC4-R after 10.7 times dilution. The supernatant of GFP transfected cells was not able to activate the MC4R (Figure 1D,E).

### Localization of flAgRP and VWF-AgRP_83-132 _in primary neurons

**Figure 2:**
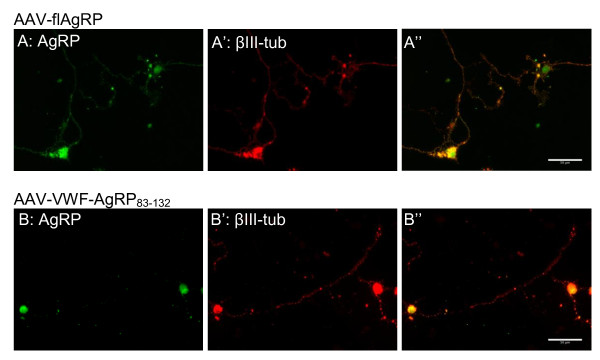
**Fluorescent immunostaining showing the localization of flAgRP versus VWF-AgRP_83-132 _**. Primary cortical neurons were infected with AAV-flAgRP(A) or AAV-VWF-AgRP_83-132 _(B) at a multiplicity of infection of 10.000. Seventy hours after infection neurons were fixed and immunostained for AgRP (green) and βIII-tubulin (red). "shows the overlay of green and red signals.

We compared the localization of AgRP in primary cortical neurons which were infected with AAV-flAgRP or AAV-VWF-AgRP_83-132 _to check if AAV-VWF-AgRP_83-132 _entered the regulated secretion pathway. Three days after infection the primary cortical neurons were stained for AgRP. In addition, β-tubulin-III was used to stain the cytoskeleton of neurons and DAPI to identify the cell nucleus. Neurons transduced with AAV-flAgRP, which contained the endogenous signal peptide to target AgRP into the regulated secretory pathways, showed AgRP immunostaining in their cell bodies and their branches. In contrast, AAV-VWF-AgRP_83-132 _transduced neurons only showed AgRP immunostaining in the cell bodies and almost no staining in the branches (Figure 2). We did not observe GFP signal without GFP immunostaining.

### Behavioral effects of AAV-VWF-AgRP_83-132 _

**Figure 3:**
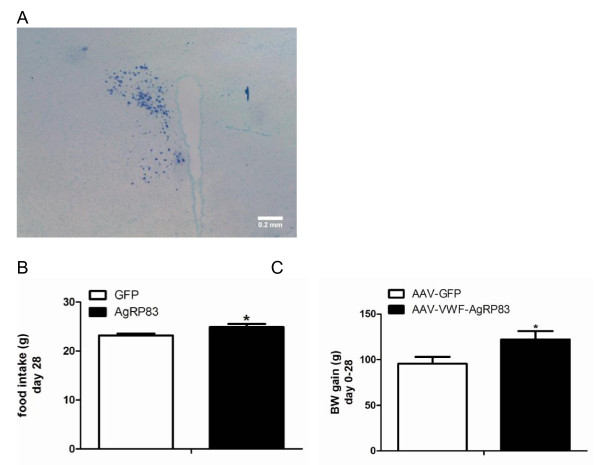
***In vivo *effects of AAV-VWF_AgRP_83-132 _on energy balance**. In situ hybridization showing unilateral transduction of the PVN by 1 μl, containing 1 x 10^9 ^g.c., of AAV-VWF-AgRP_83-132 _(A). AAV-VWF-AgRP_83-132 _increased food intake (B) and BW gain (C) compared to AAV-GFP. Data at 28 days post injection (N = 3).

To further prove the functionality of AAV-VWF-AgRP_83-132 _, this virus was also injected in the PVN of rats. One μl containing 1 × 10^9 ^genomic copies of AAV-VWF-AgRP_83-132 _or AAV-GFP (as control) was injected in the PVN (Figure 3A). In situ hybridization against GFP showed that the PVN was transduced (unilaterally). Overexpression of AAV-VWF- AgRP_83-132 _increased daily food intake when compared to titer matched AAV-GFP injected rats (p = 0.049) (Figure 3B). In addition, the AAV-VWF-AgRP_83-132 _rats showed a significant increase in body weight gain compared to AAV-GFP rats (p = 0.0032) from day 0 until day 28 post injection (Figure 3C).

## Discussion

We here successfully demonstrate a novel approach to locally overexpress a individual neuropeptide rather than a whole neuropeptide precursor. This was achieved by fusing a neuropeptide minigene to the VWF signal peptide in a rAAV vector. We demonstrated that the neuropeptide minigene was released from the cell body and did not enter the regulated secretory pathway *in vitro*. In addition, we showed that the delivery of the neuropeptide minigene, using AAV, to neurons of the PVN resulted in an increased food intake and body weight. This demonstrated that this method to deliver AgRP locally in the brain was effective.

Previous studies have shown that specific signal peptides could route a protein to the regulated pathway. Fusion of GFP to NPY or insulin signal sequence was sufficient to route GFP to regulated secretory granules and resulted in GFP secretion at axon terminals [28,29]. In addition, other groups have already showed that a secretory signal peptide can be used to direct constitutive peptide secretion from an AAV vector in the rat brain [30,31]. In this study we showed that it is also possible to route peptides into the constitutive secretion pathway resulting in secretion by the cell body, through the use of the VWF signal peptide and an AgRP fragment that lacked the N terminal part. We replaced the endogenous signal peptide, N-terminal part and cleavage site for a VWF signal peptide and a canonical proteolytic cleavage site for furin. Normally the VWF is processed in endothelial cells, however AAV transduces mainly neurons. Therefore, after the VWF signal peptide a furin recognition site (RKRR) was inserted, this to ensure that the VWF signal peptide was cleaved from AgRP_83-132 _in neurons. Probably the removal of the N-terminal part of the AgRP gene has resulted to constitutive secretion, since it is suggested that the N-terminal part of prohormone neuropeptides in involved in the sorting to the secretory or constitutive pathway. To obtain long term expression of the neuropeptide minigenes we embedded these DNA constructs in an rAAV vector. During our study a paper was published which described a method to deliver two gene products, GFP and galanin, from one rAAV vector [32]. They placed a furin sequence between the two genes and the first gene was fused to a fibronectin secretory signal peptide to establish constitutive secretion. This confirmed the feasibility of using furin as a cleavage site.

First we investigated if the vectors with the VWF signal peptide are secreted into the medium and compared this with a vector with its endogenous signal peptide.

The MC4R_CRE-LacZ assay showed that VWF-AgRP_83-132 _and VWF-αMSH1 and VWF-αMSH4 were secreted by 293T cells into the culture medium. The secreted peptides were also biologically active since the medium could, respectively, inhibit or activate the MC4R. The medium of cells transfected with flAgRP vector, containing the endogenous AgRP signal peptide, also inhibited the MC4R. However, the inhibition by flAgRP was stronger than the inhibition caused by medium from VWF-AgRP_83-132 _transfected cells. After the MC4R assay we determined the amount of protein secreted in the medium of the cells. This revealed that there was 18.2 fold less AgRP immunoreactivity in the medium of VWF-AgRP_83-132 _transfected cells than in the medium of flAgRP. The difference in potency between VWF-AgRP_83-132 _and flAgRP to antagonize NDP-MSH, 0.5 versus 1.4 nM respectively, is in the range which we expected, since a study by Creemers et. al. showed that AgRP_83-132 _was 6.1 fold more potent than flAgRP in inhibiting the MC4R [22]. Thus, given the amount of peptide release (18.2 fold less secretion of VWF-AgRP_83-132 _than flAgRP) and potencies (VWF-AgRP_83-132 _approximately 3 times less potent than flAgRP) our results are in agreement with the study by Creemers et al. that VWF-AgRP_83-132 _is 6 times more potent than flAgRP.

Medium from cells transfected with AAV-VWF-αMSH4 was more potent in activating MC4R_CRE-LacZ than medium from AAV-VWF-αMSH1 transfected cells. Medium from GFP transfected cells was not able to stimulate MC4R_CRE-LacZ. These data are in agreement with previous data [33].

In addition, we compared the localization of flAgRP and VWF-AgRP_83-132 _in primary neuronal cultures. These results showed that flAgRP was expressed in the cell body and in the projections and that VWF-AgRP_83-132 _was only expressed in the cell body and not in the projections. These results were in agreement with the expectation: peptides coupled to the VWF signal peptide and lacking the N-terminal part of AgRP entered the constitutive secretion pathway and the one coupled to the endogenous AgRP signal peptide plus N-terminal part entered the regulated secretion pathway in axons.

As a proof of principle we showed that unilateral overexpression with the VWF-AgRP_83-132 _AAV vector in a target area of AgRP neurons, the PVN, increased food intake. This showed that the VWF-AgRP_83-132 _is also functional *in vivo*.

## Conclusions

This study showed that it is possible to overexpress a single neuropeptide derived from a large precursor and establish long term release, local to the transduced area, through the use of a VWF signal peptide.

## Methods

### Cell lines and constructs

Human embryonic kidney (HEK) 293T cells were maintained at 37°C with 5% CO_2 _in Dulbecco's modified Eagles medium (DMEM) supplemented with 10% fetal calf serum (FCS), 2 mM glutamine, 100 units/ml penicillin, 100 units/ml streptomycin and non-essential amino acids.

**Figure 4:**
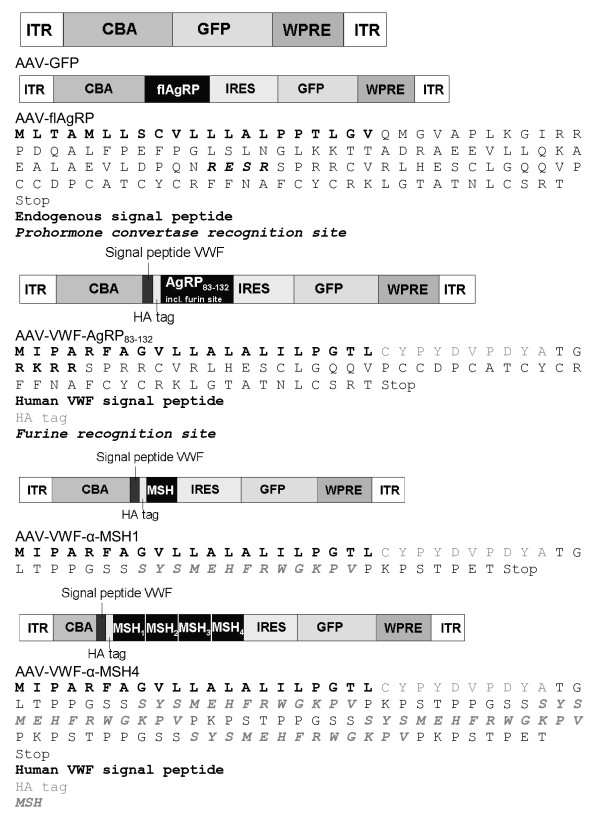
**Vectors used in the study**.

pAAV-GFP with AAV2 inverted terminal resolutions sites, was previously described [34] and was a kind gift from M. Sena-Esteves. pAAV-flAgRP-ires-GFP (Figure 4) was constructed by cloning the full length mouse Agouti-related protein (AgRP) cDNA in pIRES2-EGFP (Clontech). Subsequently, AgRP-ires-EGFP was isolated and ligated in pAAV-GFP, after removing GFP from the AAV plasmid. AgeI-furin-AgRP_83-132 _-EcoRI was made by PCR on AgRP-ires-GFP with the following primers: Agrp83f: 5' gatccaaccggtcgcaagcgtcgttctccgcgtcgctgtgtaa and ires reverse long: 5' cggcttcggccagtaacgttaggggggggggagggaga. The PCR fragment was subsequently digested with AgeI and EcoRI and ligated into AgeI- EcoRI digested pCMV-VWF-αMSH4-ires-GFP resulting in pCMV-VWF-AgRP_83-132 _-ires-GFP. pCMV-VWF-αMSH1-ires-GFP and pCMV-VWF-αMSH4-ires-GFP were described elsewhere [33]. The three pCMV-VWF-neuropeptide-ires-GFP vectors were digested with AfeI-BsrGI and the VWF-neuropeptide-ires-GFP parts were isolated and inserted in pAAV-GFP, resulting in pAAV-VWF-AgRP_83-132 _, pAAV-VWF- αMSH1 and pAAV-VWF-αMSH4 (Figure 4). Sequences were verified by sequence analysis.

The pDP1 helper plasmid used to produce AAV vectors has been described [35] and was obtained from Plasmid factory (Bielefield, Germany).

### LacZ reporter gene assay

Activation of the MC4R was determined using LacZ as a reporter gene [36]. DNA was transfected into cells with polyethylenimine [37]. Fifty nanograms of human MC4R and 10 μg of cAMP-responsive element (CRE)-LacZ were transfected into 293T cells on 10cm dishes. Another five 10cm dishes with 293T cells were transfected with 10 μg of respectively pAAV-GFP, pAAV-flAgRP, pAAV-VWF-AgRP_83-132 _, pAAV-VWF-αMSH1 and pAAV-VWF-αMSH4. One day after transfection the MC4R_CRE-LacZ cells were transferred to poly-L-lysine coated 96-wells plates. Three days after transfection supernatant of 10cm dishes was removed and used for LacZ reporter assay. At this day the medium was removed from MC4R_CRE_LacZ transfected cells and replaced with assay buffer (DMEM containing 0.2% BSA, 25 mM hepes and 30 μg/ml aprotinin) supplemented with NDP-MSH alone or in combination with conditioned supernatants of cells transfected with flAgRP, VWF-AgRP_83-132 _or GFP. In addition only forskolin was added to other wells as a positive control for CRE-LacZ transfection and the subsequent colometric reaction. The medium of another plate with MC4R_CRE-LacZ transfected cells was replaced with assay buffer containing serial dilutions of conditioned medium from cells transfected with αMSH1, αMSH4 or GFP. As assay controls only NDP-MSH or forskolin were added to other wells of this plate. After 5-6 hour incubation at 37°C the assay medium was removed and replaced by 40 μl lysisbuffer (phosphate buffered saline (PBS) containing 0.1% Triton-X-100). The plates were stored at -20°C. After thawing of the plates, 80 μl of substrate (0.1 M phosphate buffer, pH7.4, containing 1.6 g/l *o*-nitrophenyl β-D-galactopyranoside (ONPG, Invitrogen, the Netherlands), 67.5 mM β-mercaptoethanol (Merck, Germany) and 1.5 mM magnesium chloride) was added to each well. Absorbance at 405 nm was measured in a Victor^2 ^microplate reader (PerkinElmer, Brussels, Belgium).

### Spot blot

To determine the amount of protein secreted into the medium of 293T cells transfected with flAgRP, VWF-AgRP_83-132 _and GFP a spot blot was performed. A serial dilution of the medium was made in water. The first dilution was 20 times followed by 5 times dilutions. Subsequently 1 μl of undiluted and diluted conditioned medium was spotted on Hybond-C extra. The blot was dried and blocked for 10 minutes in PBS containing 0.05% Tween-20 (PBS-T) and 5% milk. Afterwards the blot was incubated with a rabbit-AgRP antibody (1:1.000; a kind gift of G. Barsh) in PBS-T for 90 minutes, washed three times 10 minutes with PBS-T and incubated for 60 minutes with goat-anti-rabbit horse-radish peroxidase (1:20.000) in PBS-T. After three washes with PBS-T and one wash with PBS the spots were developed with SuperSignal West Dura extended duration substrate (Thermo scientific) and exposed to a CL-film.

### AAV production

Each AAV production was performed with 15 dishes of 80-90% confluent 293T cells at day of transfection. Two hours before transfection, the 10% FCS-DMEM was replaced with 2% FCS-DMEM. The transfections were performed with polyethylenimine (PEI) as described by Reed S.E. et al. [37]. The pAAV plasmids were co-transfected helper plasmid pDp1 in a molar ratio of 1:1. The transfection mix remained on the cells until the next day, then the 2% FCS-DMEM was refreshed. The purification was performed as described by Zolotukhin et al. [38]. The titer, in genomic copies per ml (g.c./ml), was determined by qPCR with sybergreen mix in a LightCylcer (Roche) [39]. The qPCR primers were designed to detect BGHpolyA and were BGHpolyA_F: 5' CCTCGACTGTGCCTTCTAG; BGHpolyA_R: 5' CCCCAGAATAGAATGACACCTA. The titers were AAV-GFPl 1 × 10^13 ^g.c./ml; AAV-flAgRP 1.7 × 10^14 ^g.c./ml AAV-VWF-AgRP_83-132 _3 × 10^12 ^g.c./ml; AAV-αMSH4 2.7 × 10^14 ^g.c./ml. To obtain titer matched AAV preps, preps were diluted in PBS.

### Animals

All experimental procedures were approved by the Committee for Animal Experimentation of the University of Utrecht (Utrecht, The Netherlands).

C57BL/6 mice were obtained from Charles River. Timed-pregnant mice were killed by means of cervical dislocation. The morning on which a vaginal plug was detected was considered embryonic day 0.5 (E0.5).

Male Wistar rats, weight ranging from 220-250 g, were purchased from Charles River. All rats were individually housed in filtertop cages with *ad libitum *access to food (CRM pellets; Special Diet Services, Whitham, Essex, UK) and water. Animals were kept in a temperature- and humidity-controlled room (21 ± 2°C) with a 12 h light/dark cycle (lights on at 7:00 A.M.).

### Primary cortical neuron cultures

Cerebral cortices of E16.5 mouse embryos were dissected and dissociated by incubation with 0.25% trypsin at 37°C for 10-15 minutes, followed by tissue trituration with a fire-polished pasteurs pipet in DMEM containing 10% FCS and 20 μg/ml DNAseI. After dissociation, the medium was removed and cells were resuspended in Neurobasal medium containing B27 supplement. Dissociated cells were plated at a density of 12.000 cells per well on poly-D-Lysin- (100 μg/ml; Sigma, Zwijndrecht, The Netherlands) and laminin- (40 μg/ml; Invitrogen, Breda, The Netherlands) coated glass coverslips in 24 well plates. Neuronal cultures were maintained in a humidified incubator at 37°C and 5% CO_2 _. After two days in culture, half of the medium was replaced with fresh maintenance medium (Neurobasal medium with B27). Two hours later neurons were infected with the different AAV vectors at multiplicity of infection 10.000 (thus 1.2 × 10^8 ^g.c. of AAV vector added per well). Seventy-two hours after infection neurons were washed twice with PBS, fixed with 4% paraformaldehyde for 30 minutes and stored in PBS at 4°C.

### Immunohistochemistry

Fixed neurons were washed twice with PBS. After 30 minutes incubation in block-t (PBS containing 1% FCS and 0.1% Triton-X-100) neurons were incubated overnight with block-t containing rabbit anti-AgRP antibody (1:1000, a kind gift from G. Barsh) and mouse anti-βIII-tubulin (1:3000, Sigma) at 4°C. The next day neurons were washed 3 times with PBS-t (PBS containing 0.1% Triton-X-100) and incubated for 60 minutes with secondary antibodies (goat-anti-mouse alexa555 (1:500) and goat-anti-rabbit alexa488 (1:500) (Invitrogen) in block buffer (PBS containing 1% FCS). Afterwards neurons were washed 3 times with PBS, incubated with DAPI (1:3000, Sigma) for 5 minutes, washed 3 times with PBS and mounted in 90% glycerol in PBS.

### In vivo AAV injections

Six rats were anesthetized with fentanyl/fluanisone (Hypnorm, Janssen Pharmaceutica, Beerse, Belgium, 0.1 ml/100 g intramuscular). Carprofen (Rimadyl^®^, Pfizer Animal Health, Capelle a/d Ijssel, The Netherlands, 0.01 ml/100 g subcutaneous) was administered as pain medication, before surgery and for 2 days after. Subsequently, the rats were injected unilaterally in the paraventricular nucleus (PVN, coordinates AP -1.80, ML +1.70, DV -8.10, angle 10 degrees) with 1 μl of AAV-GFP or AAV-VWF-AgRP_83-132 _. The injected volume contained 1 x 10^9 ^g.c. of AAV vector and was delivered at a rate of 0.2 μl/minute. Subsequently the injection the needle remained in the injection site for 10 minutes. Four weeks after injection the rats were decapitated, the brains were removed, quickly frozen on dry-ice and stored at -80°C until they were sectioned on a cryostat (Leica, The Netherlands) at 20 μm, in series of 10. Serie 1 was used for *in situ *hybridization with GFP-dig labeled mRNA probe to show the transduction area of the AAV vectors and serie 2 for a Nissl staining to show the overall morphology. The body weight and food intake data of animals with correct injection sites, as determined with GFP ISH, were used for behavioral analysis.

### In situ hybridization

*In situ *hybridization was performed with a 720 basepair long digoxigenin (DIG)-labeled eGFP riboprobe (antisense to NCBI gene DQ768212) as described by Schaeren-Wiemers and Gerfin-Moser [40] with small modifications in the fixation procedure and hybridization temperature. Sections were fixed in 4% PFA for 20 minutes and hybridization was performed at 72°C. After DIG in situ hybridization, slides were counterstained with 0.5% methyl green, quickly dehydrated in ethanol, cleared in xylene and mounted using Entellan.

### Data analysis

Data were analyzed with GraphPad Prism (GraphPad Software Inc., California). Competition curves were fitted from duplicate data points using sigmoidal dose response curve with variable slope.

Body weight gain and food intake were tested with two sided *t*-test.

## Authors' contributions

MWAdB constructed the AAV vectors, produced and purified them with the help of KGM. MWAdB performed and analyzed LacZ reporter assays, spot blot, immunohistochemstry and in situ hybridizations; discussed the results and prepared the manuscript. MADB and MCML performed the animal work including stereotaxic injections. DMAvdH and RJP provided the primary cortical cultures to MWAdB to transduce them and have discussed the results. RAH participated in experimental design and supervised the experiments, discussed results, corrected the manuscript and provided financial support. All authors have read and approved the final manuscript.
